# Specific gut microbiota features and metabolic markers in postmenopausal women with obesity

**DOI:** 10.1038/nutd.2015.9

**Published:** 2015-06-15

**Authors:** L K Brahe, E Le Chatelier, E Prifti, N Pons, S Kennedy, T Hansen, O Pedersen, A Astrup, S D Ehrlich, L H Larsen

**Affiliations:** 1 Department of Nutrition, Exercise and Sports, University of Copenhagen, Frederiksberg C, Denmark; 2 Metagenopolis, Institut National de la Recherche Agronomique, Jouy-en-Josas, France; 3 Novo Nordisk Foundation Centre for Basic Metabolic Research, University of Copenhagen, Copenhagen, Denmark

## Abstract

**Background:**

Gut microbial gene richness and specific bacterial species are associated with metabolic risk markers in humans, but the impact of host physiology and dietary habits on the link between the gut microbiota and metabolic markers remain unclear. The objective of this study was to identify gut metagenomic markers associated with estimates of insulin resistance, lipid metabolism and inflammation in obesity, and to explore whether the associations between metagenomic and metabolic markers persisted after adjustment for body fat, age and habitual dietary intake.

**Methods:**

Faecal DNA from 53 women with obesity was analysed through quantitative metagenomic sequencing and analysis, and a systematic search was performed for bacterial genes associated with estimates of insulin resistance, inflammation and lipid metabolism. Subsequently, the correlations between metagenomic species and metabolic markers were tested by linear regression models, with and without covariate adjustment.

**Results:**

One hundred and fourteen metagenomic species correlated with metabolic markers (*P*<0.001) including *Akkermansia muciniphila, Bilophila wadsworthia, Bifidobacterium longum* and *Faecalibacterium prausnitzii*, but also species not previously associated with metabolic markers including *Bacteroides faecis* and *Dorea longicatena*. The majority of the identified correlations between bacterial species and metabolic markers persisted after adjustment for differences in body fat, age and dietary macronutrient composition; however, the negative correlation with insulin resistance observed for *B. longum* and *F. prausnitzii* appeared to be modified by the intake of dietary fibre and fat, respectively.

**Conclusions:**

This study shows that several gut bacterial species are linked to metabolic risk markers in obesity, also after adjustment for potential confounders, such as long-term diet composition. The study supports the use of gut metagenomic markers for metabolic disease prediction and warrants further investigation of causality.

## Introduction

The human gut hosts trillions of microbes with a collective genome (the gut microbiome) that consists of nearly 10 million genes,^1^ which exceeds more than 400-fold the size of the human genome that consists of approximately 23 000 genes.^2^ This vast gut microbiome provides the human host with vital functions that affect nutritional efficiency and overall health.^3^ Metagenomic studies have suggested that individuals with a metabolic risk profile are characterized by low gut microbiome richness,^4, 5^ and negative correlations have been found between bacterial gene count and metabolic risk markers in the present cohort of postmenopausal women with obesity.^6^ Additionally, studies have shown that microbial gene markers correlate better with type-2 diabetes (T2D) than both anthropometric risk markers^7^ and common variation in the human genome.^8^ Thus, the gut microbiome has been proposed as a marker for early identification of individuals at increased risk of obesity-related metabolic diseases.^5^

The gut microbiota develops immediately after birth influenced by delivery mode, early nutrition and host genotype,^3^ and within the first 3 years of life, the microbial diversity reaches a level similar to adulthood.^9^ The composition of the adult gut microbiota is considered overall stable.^10^ Yet, several factors modulate the adult gut microbiota including age, antibiotics and diet.^3^ A modulating effect of dietary intake on the gut microbiota has been shown by the impact of long-term dietary habits,^9, 11, 12^ changes in macronutrient composition^13^ and shifts in intake of animal- *versus* plant-based diets.^14^ In addition, the relative abundance of bacterial species and the microbial diversity vary with the physiological state of the host, shown by the altered gut microbiota in pregnancy,^15^ inflammatory bowel disease,^16^ obesity,^17^ T2D,^8^ atherosclerosis,^18^ non-alcoholic fatty liver disease^19^ and liver cirrhosis.^20^

The impact of host physiology and dietary habits on the link between the gut microbiota and metabolic markers remains uncertain. One study that identified an inverse correlation between gut microbial gene richness and metabolic risk in humans also recognized that individuals with low bacterial richness (<480 000 bacterial genes) consumed less fruit, vegetables and fish, compared to individuals with high bacterial richness.^4^ In addition, large differences have been reported in the prevalence of low bacterial richness in obese individuals at increased metabolic risk; ranging from 8%^6^ to 40%,^4^ which could be due to variation in dietary habits.

The objective of this study was to identify gut metagenomic markers associated with insulin resistance, lipid metabolism and inflammation in obese women, and to explore whether the associations between metagenomic and metabolic markers persisted after adjustment for differences in age, body fat and habitual dietary intake.

## Subjects and methods

### Study design and participants

This study includes baseline data from 53 participants who completed a dietary intervention with intake of *Lactobacillus paracasei* F19, flaxseed mucilage or placebo over 6 weeks.^6^ The study took place at the Department of Nutrition, Exercise and Sports (NEXS), Faculty of Sciences, University of Copenhagen, Denmark, from September 2011 to September 2012. Written informed consent was obtained from all participants; the study was approved by the Ethical Committee for the Capital Region of Denmark (journal H-3-2011-067) in accordance with the Helsinki II Declaration and registered at ClinicalTrial.gov (NCT01433120). The study included postmenopausal women with a body mass index (BMI) of 30–45 kg m^−2^. Exclusion criteria were gastrointestinal diseases, chronic diseases as type-1 diabetes or liver cirrhosis, medically-treated T2D or dyslipidaemia, intake of antibiotics, supplementary pro- or prebiotics or high quantities of fermented foods (>400 g day^−1^) in the previous months.

### Anthropometric and biochemical measurements

Anthropometric and biochemical measurements were obtained after an overnight fast. Body composition was assessed by dual-energy X-ray absorptiometry (DXA) (iDXA, Lunar Radiation Co., Madison, WI, USA), BMI (kg m^−^^2^), waist circumference and intra-abdominal adipose tissue (IAAT cm^2^: −208.2+4.62 (sagittal diameter, cm)+0.75 (age, years)+1.73 (waist, cm)+0.78 (trunk fat %)).^21^ An oral glucose tolerance test was performed and insulin sensitivity was assessed by plasma glucose, serum insulin and C-peptide at the fasting and stimulated state. Insulin resistance was estimated by homeostatic model assessment of insulin resistance (HOMA-IR) (F-glucose (mmol l^−1^) × F-insulin (mU l^−1^)/22.5).^22^ Data from the oral glucose tolerance test were evaluated by area under the curve (AUC) analysis and by Matsudas index (10 000 / √ (F-glucose (mg dl^−1^)  × F-insulin (mU l^−1^) × (mean oral glucose tolerance test glucose × mean oral glucose tolerance test insulin)).^23^

Inflammatory status was assessed by white blood cell (WBC) count, fasting concentration of plasma high sensitive C-reactive protein (hsCRP), lipopolysaccharide-binding protein (LBP), cluster of differentiation 14 (CD14), and serum concentrations of the liver enzymes aspartate aminotransferase (AST) and alanine aminotransferase (ALT). Lipid metabolism was assessed by fasting concentrations of serum total cholesterol, low-density lipoprotein (LDL) cholesterol, high-density lipoprotein (HDL) cholesterol, triglycerides (TAG) and plasma concentrations of free fatty acids (FFAs).

Blood for analysis of CD14 and FFA was drawn in iced tubes pre-coated with EDTA. Blood for analysis of AST and ALT was collected in plain tubes. Blood samples were centrifuged for 10 min at 2500 *g* at 4 °C and kept at −80 °C until analyses were performed. ABX Pentra 400 (Horiba ABX, Montpellier, France) was used to analyse blood concentrations of FFA (intra- and inter-assay coefficient of variability: 1.7 and 5.3%), ALT (intra- and inter-assay coefficient of variability: 3.1 and 6.0%) and AST (intra- and inter-assay coefficient of variability: 2.7 and 5.0%). CD14 (intra- and inter-assay coefficient of variability: 6.5 and 1.7%) was analysed with a human Elisa kit (R&D Systems, Minneapolis, MN, USA). Details of all other biochemical analyses have been described elsewhere.^6^

### Dietary records

Registration of 3 days weighed dietary intake was performed within the week before the baseline visit to obtain information about habitual dietary habits. Dietary records were analysed by a registered dietician by use of a dietary software program (Dankost Pro, Copenhagen, Denmark).

### Microbiota analyses

Participants collected samples of stool within 2 days before the visit. Samples were either stored immediately at −80 °C or briefly stored in a −18 °C freezer and kept frozen during the transport to the laboratory. Total DNA from faecal samples was extracted, sequenced and analysed by quantitative metagenomics at Metagenopolis (INRA, Jouy-en-Josas, France), a detailed description of the metagenomic analysis is provided elsewhere.^6^ DNA sequencing data were generated using the SOLiD 5500xl sequencers (Life Technologies, Carlsbad, CA, USA). Primary data analyses were performed using METEOR Studio pipeline for quantitative metagenomic profiling developed at INRA MetaGenoPolis based on the iMOMi database. Reads generated from the SOLiD sequencer were trimmed to 35 bases then mapped by Bowtie software on the reference catalogue of 3.3 million genes.^16^ Microbial gene richness was measured by counting the number of genes in a given sample by use of a downsized count matrix at 11 million unique reads as previously described.^5^ The exponential of Shannon diversity index was included as a measure of alpha diversity.

A systematic search was performed for bacterial genes correlated with metabolic markers and the bacterial genes were subsequently clustered into metagenomic species (MGS) using the method based on binning co-abundant genes across all individuals samples described elsewhere.^5^ A MGS was assigned to a given genome when more than 80% of its genes matched the same genome using blastN at a threshold of 95% identity over 90% of gene length. The remaining MGSs were annotated using blastP analysis and assigned to a given taxonomical level from genus to super kingdom level if more than 80% of their genes had the same level of assignment.

### Statistical analyses

The systematic search for bacterial genes that correlated with markers for insulin sensitivity, lipid metabolism or inflammation was performed by use of Spearman's rank correlation coefficient. Correction for multiple testing was performed by the Benjamin–Hochberg method with the false discovery rate at 5%. Correlations between a MGS and metabolic or dietary markers are reported by Spearman's Rho (*r*) and *P*-values. In addition, correlations between bacterial species and metabolic markers were tested by linear regression analyses, with and without adjustment for differences in age, body fat percentage and when applicable; the dietary component with the strongest impact. Results from the regression models are reported by Pearson's correlation coefficient (*r*) and *P*-values (both adjusted and non-adjusted). Log transformation was applied to non-normally distributed variables. Statistical significance was attested at a two-sided *P*-value of <0.05 and trend towards significance was attested at a *P*-value between 0.5 and 0.1. Analyses were performed using MeatOMiner R package (developed at Metagenopolis, INRA, France) and JMP version 9.0.2 (SAS Institute Inc, Cary, NC, USA).

## Results

Baseline data for the participants are reported in Table 1. Of the 53 participants, 36 (68%) could be classified as metabolic unhealthy, defined as the presence of the metabolic syndrome,^24^ pre-diabetes or T2D.^25^

### Gut bacteria associated with metabolic markers

A total of 55 069 bacterial genes correlated with metabolic markers (*P*<0.001). In all, 31 134 genes (57%) could be clustered in 114 MGSs each with more than 50 microbial genes. Eighty-two MGSs were assigned to a bacterial phylum and 32 MGSs were unknown. Of the known MGSs; 55 were identified to species level. The MGS abundance signals within the individuals, the size of the MGS and the taxonomy data are given as Supplementary information.

**Figure 1:**
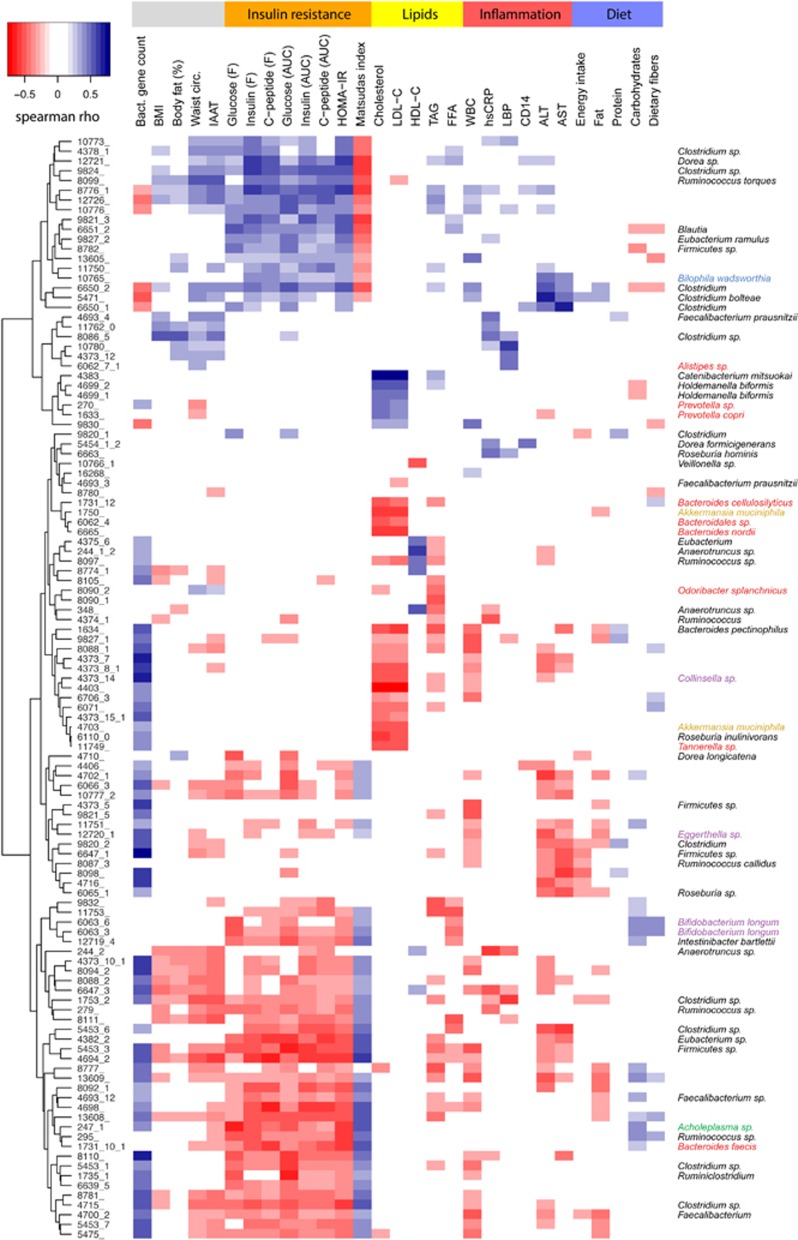
Heatmap of correlations between MGS and markers for insulin resistance, lipid metabolism, inflammation, body composition, dietary intake and bacterial gene count. The heatmap reports significant Spearman's rank correlations (*P*<0.001) observed between 114 MGS signals (rows) and 29 different markers (columns) using Euclidean distance metric and complete linkage method. Markers are grouped in five categories given in the top of the figure: insulin resistance, lipid metabolism, inflammation, diet and others. MGS names are shown on the left of the figure and their taxonomical annotation at the species or genus level is given on the right, coloured according to the phylum: Firmicutes in black, Bacteroidetes in red, Actinobacteria in purple, Proteobacteria in blue, Verrucomicrobia in gold and Tenericutes in green. The strength of the correlation is coloured according to the colour key in the upper left corner of the figure. ALT, alanine aminotransferase; AST, aspartate aminotransferase; AUC, area-under-the-curve; BMI, body mass index; CD14, cluster of differentiation 14; F, fasting; FFAs, free fatty acids; HDL-C, high-density lipoprotein cholesterol; hsCRP, high sensitive C-reactive protein; HOMA-IR, homeostatic model assessment of insulin resistance; IAAT, intra-abdominal adipose tissue; LBP, lipopolysaccharide-binding protein; LDL-C, low-density lipoprotein cholesterol; TAG, triglycerides; Waist cir.; waist circumference; WBC, white blood cells.

The MGSs that were associated with insulin sensitivity, inflammatory markers and lipid metabolism are presented in Figure 1 and Supplementary Tables S1-S3. *Bacteroides faecis*, *Intestinibacter bartlettii*, *Bifidobacterium longum*, *F. prausnitzii A2-165* and *Dorea longicatena* were negatively correlated with markers for insulin resistance, whereas *Ruminococcus torques*, *Clostridium bolteae, Eubacterium ramulus* and *Bilophila wadsworthia* were positively correlated (Supplementary Table S1).

*Bacteroides pectinophilus* was negatively correlated with inflammatory markers, whereas *C. bolteae*, *Dorea formicigenerans*, *B. wadsworthia*, *Roseburia hominis* and *F. prausnitzii* *SL3/3* were positively correlated (Supplementary Table S2).

*Odoribacter splanchnicus*, *B. pectinophilus*, *Bacteroides cellulosilyticus*, *Bacteroides nordii*, *Roseburia inulinivorans*, *Akkermansia muciniphila, F. prausnitzii A2-165* and *B. longum* were associated with a healthy fasting serum lipid profile, defined as a positive correlation with HDL cholesterol or a negative correlation with TAG, FFAs, total- or LDL cholesterol. *Catenibacterium mitsuokai* and *Holdemanella biformis* were associated with an unhealthy fasting serum lipid profile (Supplementary Table S3).

Of the 32 unknown MGSs, 21 were associated with markers for insulin resistance (mainly negative correlations), 27 were associated with inflammatory markers (mainly negative correlations with WBC) and 20 were associated with markers for lipid metabolism (mainly negative correlations with total cholesterol and TAG) (data not shown).

### Link between bacterial species and metabolic markers after covariate adjustment

The bacterial species that were linked to habitual dietary intake (total energy, macronutrient composition, dietary fibres) by Spearman's rank correlation are presented in Figure 1 and Supplementary Table S4. Species associated with a healthy metabolic profile (Supplementary Tables S1-S3) were generally negatively correlated with intake of dietary fat and positively correlated with intake of carbohydrates, specifically dietary fibres (Supplementary Table S4), while the opposite was observed for species associated with an unhealthy metabolic profile. Microbial gene richness and alpha diversity correlated positively with overall protein intake (Supplementary Table S4), but when the types of protein were considered, the correlations between proteins from meat sources and microbial gene richness and alpha diversity tended to be negative (*r*=−0.25, *P*=0.08 and *r*=−0.27, *P*=0.05, respectively), while the correlations with proteins from sources other than meat, including vegetables and fish were positive (*r*=0.27, *P*=0.06 and *r*=0.35, *P*=0.01, respectively).

The majority of the associations between bacterial species and metabolic markers persisted when tested by use of multiple linear regression models with adjustment for differences in body fat, age and associated dietary components (Supplementary Table S4). The adjusted and non-adjusted results for these persistent associations between bacterial species and relevant markers for insulin resistance and dyslipidaemia are reported in Tables 2 and 3, respectively.

Some identified associations between metagenomic and metabolic markers were affected by the statistical adjustment; the negative correlation between *B. longum* and markers for insulin resistance disappeared after adjustment for intake of carbohydrates, but the negative correlation with fasting glucose persisted (Table 2). In addition, the negative correlation between *F. prausnitzii A2-165* and markers for insulin resistance disappeared after adjustment for intake of fat.

## Discussion

This study shows that several gut bacterial species are linked to metabolic risk markers in obesity after adjustment for the potential confounders age, body fat and long-term diet composition, supporting the proposed use of gut metagenomic markers for metabolic disease prediction and stratification,^5, 7, 8^ and suggesting that gut bacteria may have a causal role in the development of obesity-related metabolic disease.

The species *A. muciniphila*, *B. cellulosilyticus*, *B. faecis*, *B. nordii*, *B. pectinophilus*, *I. bartlettii*, *D. longicatena*, *O. splanchnicus* and *R. inulinivorans* were all negatively associated with markers for insulin resistance or dyslipidaemia, also after covariate adjustment, suggesting a probiotic potential of these bacteria. Some of these species have been linked to metabolic health previously; *B. pectinophilus* has been found to be more abundant in lean individuals, compared with obese,^5^ and the butyrate-producing *R. inulinivorans*^26^ has been found to be more abundant in healthy individuals, compared with individuals with T2D.^8^ *A. muciniphila*, which was found to be negatively associated with serum total and LDL cholesterol in the present study, is considered as the most abundant mucolytic bacteria in healthy humans.^27^ A reduced abundance of *A. muciniphila* could reflect a thin mucus layer and thus an impaired gut barrier function with increased translocation of pro-inflammatory bacterial toxins potentially leading to metabolic disturbances. This mechanism is supported by the reduced abundance of *A. muciniphila* observed in patients with inflammatory bowel disease.^28^ Additionally, *A. muciniphila* has been shown to alleviate metabolic disturbances in mice on a high-fat diet, probably due to prevention of a high-fat diet-induced decrease in the mucus layer and thus bacterial toxin translocation.^29, 30^ Previously, *A. muciniphila* has been found to be more abundant in the gut microbiota of women with normal weight gain, compared with excessive weight gain during pregnancy,^31^ and in normal weight, compared with overweight children.^32^ However, it cannot be excluded that these associations were affected by a lower intake of dietary fat in the normal weight individuals, as these studies did not correct for the negative correlation that seem to exist between dietary fat intake and gut abundance of *A. muciniphila* (Supplementary Table S4). On the contrary, a metagenomic study identified increased abundance of *A. muciniphila* in patients with T2D, compared with healthy individuals.^8^ However, discrepancy between results could be explained by differences between study populations, as the study included patients with diagnosed T2D who might have been in treatment with anti-diabetic and/or lipid-lowering medication as opposed to the present study, where T2D and/or dyslipidaemia were incident. This is supported by studies in mice where the antidiabetic drug metformin causes an increase in the abundance of *A. muciniphila*.^30, 33^ This theory might also explain why Qin *et al.*^8^ found increased abundance of *B. cellulosilyticus* and *O. splanchnicus* in patients with T2D, whereas we find these two species to be associated with a healthy lipid metabolism. The link to a healthy host metabolism for *B. faecis*, *B. nordii*, *I. bartlettii* and *D. longicatena* has not been reported previously, and there are no recognizable characteristics of these species that may explain the associations.

*B. wadsworthia*, *C. bolteae*, *C. mitsuokai*, *E. ramulus*, *H. biformis* and *R. torques* were all positively associated with insulin resistance or dyslipidaemia, even after covariate adjustment. This is a novel finding for the species *C. mitsuokai, E. ramulus* and *H. biformis*, whereas the link to insulin resistance observed for *B. wadsworthia* and *C. bolteae* supports the increased abundance of these bacteria identified in patients with T2D.^8^ The abundance of *B. wadsworthia* has also been shown to increase after short-term intake of an animal-based high-fat diet.^14^ However, there were no association between the abundance of *B. wadsworthia* and habitual dietary intake of meat, total fat, monounsaturated-, polyunsaturated- or saturated fatty acids in this study. The mucin-degrading *R. torques* has been shown to be more abundant in the gut microbiota of patients with inflammatory bowel disease^28^ and in individuals with low microbiome gene richness.^5^ It is possible that the positive association with insulin resistance identified for *R. torques* can be explained by a harmful effect of this bacterium on the gut barrier, leading to metabolic endotoxaemia.^34^

The results indicate that intake of protein from other dietary sources than meat is positively correlated with bacterial gene richness and diversity. This might provide an explanation for the discrepancies in numbers of individuals with low bacterial richness identified between obese cohorts^4, 5, 6^ as the low prevalence of individuals with low gene count in our study could be due to a higher habitual intake of non-meat protein.

Previous studies in humans have reported a higher abundance of *Bifidobacterium* including *B. longum* in healthy individuals, compared with individuals with obesity^35, 36^ and T2D,^37^ and increased abundance of *Bifidobacterium* has been linked to a reduction in inflammatory markers and an improvement in glucose homeostasis and lipid metabolism.^38, 39, 40^ However, we found that the negative correlation between *B. longum* and both HOMA-IR and Matsudas index disappeared after adjustment for carbohydrate intake. Although, the negative correlation with plasma glucose persisted, this suggests that beneficial effect of *Bifidobacterium* on host health also depends on dietary intake, and that beneficial associations identified between abundance of *Bifidobacterium* and metabolic health partly reflect higher intake of dietary fibres in healthy individuals. *F. prausnitzii A2-165* was negatively correlated with markers for insulin resistance, but the negative correlation between *F. prausnitzii A2-165* and markers for insulin resistance disappeared when adjusted for dietary fat intake. This suggests that the lower abundance of *F. prausnitzii* previously reported in individuals with T2D^7, 8^ could be influenced by differences in dietary fat intake. However, this cannot be confirmed as these two studies did not report dietary intake. Interestingly, we identified opposing correlations for different *F. prausnitzii* strains; *F.prausnitzii SL3/3* was positively correlated with hsCRP but was not associated with markers for insulin resistance or dietary fat intake, indicating that it is necessary to evaluate the effects of *F. prausnitzii* species at the strain level.

As most of the beneficial bacteria identified in this study appear to be stimulated by specific macronutrients (Supplementary Table S4), a potential approach towards metabolic disease prevention could combine intake of beneficial bacteria with nutrients that generate a beneficial gut environment for the bacteria and improve host health through separate mechanisms. For instance, dietary supplements with *I. bartlettii*, *B. cellulosilyticus* and *B. longum* in combination with intake of a diet high in water-soluble viscous fibres might improve glucose homeostasis and dyslipidaemia, equally by fibre-induced stimulation of the growth of these specific bacteria in the colon and by the gel forming capacity of the fibres in the gastrointestinal tract that delays gastric emptying and inhibits absorption of glucose and cholesterol.^41^

In summary, this study shows that the link between certain gut bacteria and metabolic risk markers in obesity is independent of variation in body fat, age and long-term dietary habits, supporting the use of gut metagenomic markers for metabolic disease prediction, suggesting a causal role of gut bacteria in development of obesity-related metabolic diseases and a potential for microbiota modulation as a strategy to improve host health. In addition, the study stresses the importance of a dietary focus when studying the link between the gut microbiota and metabolic markers, as it shows that the positive association with a healthy metabolic profile suggested for *B. longum* and *F. prausnitzii* appears to be modified by intake of dietary fibre and fat, respectively. Furthermore, it proposes the search for associations between dietary components and specific gut microbiota features as a strategy for the development of new synbiotic products with potential for metabolic disease prevention.

## Figures and Tables

**Table 1:** Baseline characteristics

*N*	53
Metabolic healthy (*N*)	17
Metabolic unhealthy (*N*)	36
Age (years)	60 (6)
BMI (kg m^−^^2^)	34.5 (3.8)
Body fat (%)	47.0 (3.7)
Waist circumference (cm)	104.3 (9.3)
IAAT (cm^2^)	171 (29)
	
*Insulin resistance*	
Fasting plasma glucose (mmol l^−1^)	5.8 (0.8)
Fasting serum insulin (pmol l^−1^)	93.2 (52.3)
Fasting serum C-peptide (pmol l^−1^)	843 (336)
AUC glucose (mmol l^−1^ per minute)	7.2 (1.5)
AUC insulin (pmol l^−1^ per minute)	414 (243)
AUC C-peptide (pmol l^−1^ per minute)	2777 (1020)
HOMA-IR	4.2 (2.6)
Matsudas index	2.4 (1.6)
	
*Inflammatory markers*	
WBC (10^9^ per litre)	5.5 (1.2)
Fasting plasma hsCRP (mg l^−1^)	3.6 (3.5)
Fasting plasma LBP (μg l^−1^)	19.0 (6.4)
Fasting plasma CD14 (ng ml^−1^)	1719 (269)
	
*Liver enzymes*	
Fasting serum ALT (U l^−1^)	17.3 (10.1)
Fasting serum AST (U l^−1^)	21.0 (6.6)
	
*Lipid metabolism*	
Fasting serum total cholesterol (mmol l^−1^)	6.1 (0.9)
Fasting serum HDL cholesterol (mmol l^−1^)	1.5 (0.3)
Fasting serum LDL cholesterol (mmol l^−1^)	3.8 (0.8)
Fasting serum TAG (mmol l^−1^)	1.4 (0.7)
Fasting plasma FFAs (μmol l^−1^)	647 (161)
	
*Dietary intake*	
Total energy intake (kJ day^−1^)	7572 (1797)
Protein (E%)	18.9 (3.6)
Carbohydrates (E%)	40.8 (6.8)
Dietary fibres (g day^−1^)	21.3 (6.0)
Fat (E%)	35.3 (6.3)

Abbreviations: ALT, alanine aminotransferase; AST, aspartate aminotransferase; AUC, area under the curve; BMI, body mass index; CD14, cluster of differentiation 14; E%, energy percentage; FFAs, free fatty acids; HDL, high-density lipoprotein; hsCRP, high sensitive C-reactive protein; HOMA-IR, homeostatic model assessment of insulin resistance; IAAT, intra-abdominal adipose tissue; LBP, lipopolysaccharide-binding protein; LDL, low-density lipoprotein; *N*, number of participants; TAG, triglycerides; WBC, white blood cells. Data are presented as mean (s.d.).

**Table 2:** Correlations between bacterial species and insulin resistance with and without covariate adjustment

*Species*	*Glucose (F)*	*Insulin (F)*	*HOMA-IR*	*Matsudas index*
*Negatively associated with insulin resistance*				
* Bacteroides faecis*	−0.38 (0.003)	−0.47 (<0.001)	−0.49 (<0.001)	0.48 (<0.001)
* *Adj. (age and body fat)	−0.37 (0.004)	−0.47 (<0.001)	−0.48 (<0.001)	0.50 (<0.001)
* *Adj. (diet)	−0.51 (0.015)	−0.52 (0.002)	−0.56 (<0.001)	0.57 (0.001)
* *Adj. (full model)	−0.49 (0.014)	−0.51 (0.002)	−0.54 (0.001)	0.63 (0.002)
* Bifidobacterium longum*	−0.44 (0.001)	−0.28 (0.031)	−0.34 (0.012)	0.39 (0.004)
* *Adj. (age and body fat)	−0.40 (0.011)	−0.31 (0.025)	−0.34 (0.014)	0.43 (0.004)
* *Adj. (diet)	−0.44 (0.036)	−0.38 (0.563)	−0.42 (0.359)	0.45 (0.205)
* *Adj. (full model)	−0.40 (0.089)	−0.39 (0.290)	−0.41 (0.205)	0.48 (0.094)
* Dorea longicatena*	−0.50 (<0.001)	−0.20 (0.090)	−0.28 (0.023)	0.27 (0.035)
* *Adj. (age and body fat)	−0.49 (<0.001)	−0.29 (0.031)	−0.35 (0.007)	0.40 (0.008)
* *Adj. (diet)	−0.52 (<0.001)	−0.13 (0.102)	−0.25 (0.025)	0.23 (0.033)
* *Adj. (full model)	−0.50 (<0.001)	−0.26 (0.048)	−0.32 (0.011)	0.37 (0.011)
* Intestinibacter bartlettii*	−0.37 (0.006)	−0.30 (0.023)	−0.35 (0.012)	0.37 (0.007)
* *Adj. (age and body fat)	−0.33 (0.013)	−0.40 (0.024)	−0.40 (0.010)	0.47 (0.018)
* *Adj. (diet)	−0.60 (0.008)	−0.45 (0.038)	−0.51 (0.019)	0.54 (0.010)
* *Adj. (full model)	−0.57 (0.010)	−0.54 (0.017)	−0.57 (0.009)	0.63 (0.004)
				
*Positively associated with insulin resistance*				
* Bilophila wadsworthia*	0.06 (0.369)	0.36 (0.004)	0.33 (0.008)	−0.32 (0.014)
* *Adj. (age and body fat)	0.06 (0.255)	0.41 (0.002)	0.38 (0.004)	−0.41 (0.006)
* *Adj. (diet)	0.14 (0.337)	0.34 (0.005)	0.31 (0.009)	−0.29 (0.013)
* *Adj. (full model)	0.16 (0.255)	0.39 (0.004)	0.36 (0.006)	−0.39 (0.008)
* Clostridium bolteae*	0.32 (0.012)	0.36 (0.005)	0.38 (0.003)	−0.35 (0.006)
* *Adj. (age and body fat)	0.35 (0.005)	0.40 (0.003)	0.42 (0.001)	−0.44 (0.002)
* *Adj. (diet)	0.34 (0.038)	0.39 (0.017)	0.42 (0.011)	−0.43 (0.025)
* *Adj. (full model)	0.38 (0.017)	0.43 (0.011)	0.46 (0.006)	−0.51 (0.011)
* Eubacterium ramulus*	0.40 (0.002)	0.37 (0.004)	0.42 (0.001)	−0.40 (0.002)
* *Adj. (age and body fat)	0.38 (0.003)	0.41 (0.006)	0.43 (0.002)	−0.46 (0.004)
* Ruminococcus torques*	0.17 (0.129)	0.39 (0.002)	0.39 (0.003)	−0.39 (0.003)
* *Adj. (age and body fat)	0.14 (0.115)	0.37 (0.006)	0.36 (0.007)	−0.39 (0.008)
* *Adj. (diet)	0.24 (0.313)	0.41 (0.010)	0.41 (0.014)	−0.45 (0.015)
* *Adj. (full model)	0.24 (0.294)	0.40 (0.026)	0.40 (0.030)	−0.47 (0.044)

Abbreviations: F, fasting; HOMA-IR, homeostatic model assessment of insulin resistance. Correlations are presented as Pearson's *r* with *P*-values in parentheses, with and without adjustment (adj.) for age, body fat, and when relevant; the dietary component with the strongest impact (Supplementary Table S4). Full models are adjusted for age, body fat and diet.

**Table 3:** Correlations between bacterial species and lipid metabolism with and without covariate adjustment

*Species*	*TAG*	*Cholesterol*	*HDL-C*	*LDL-C*
*Associated with a healthy lipid metabolism*				
* Akkermansia muciniphila*	−0.17 (0.120)	−0.42 (0.002)	0.14 (0.826)	−0.37 (0.005)
* *Adj. (age and body fat)	−0.32 (0.091)	−0.39 (0.002)	0.17 (0.886)	−0.33 (0.006)
* *Adj. (diet)	−0.17 (0.195)	−0.40 (0.002)	0.21 (0.900)	−0.34 (0.007)
* *Adj. (full model)	−0.32 (0.156)	−0.37 (0.002)	0.07 (0.926)	−0.29 (0.008)
* Bacteroides cellulosilyticus*	−0.37 (0.004)	−0.48 (<0.001)	−0.13 (0.781)	−0.38 (0.003)
* *Adj. (age and body fat)	−0.45 (0.002)	−0.48 (<0.001)	−0.11 (0.883)	−0.37 (0.002)
* *Adj. (diet)	−0.36 (0.007)	−0.47 (<0.001)	−0.19 (0.748)	−0.38 (0.007)
* *Adj. (full model)	−0.43 (0.003)	−0.46 (<0.001)	−0.11 (0.875)	−0.36 (0.005)
* Bacteroides nordii*	−0.24 (0.050)	−0.50 (<0.001)	0.09 (0.458)	−0.46 (<0.001)
* *Adj. (age and body fat)	−0.30 (0.049)	−0.48 (<0.001)	0.10 (0.437)	−0.44 (<0.001)
* Bacteroides pectinophilus*	−0.42 (0.001)	−0.42 (0.001)	0.12 (0.199)	−0.47 (<0.001)
* *Adj. (age and body fat)	−0.53 (<0.001)	−0.41 (<0.001)	0.25 (0.128)	−0.46 (<0.001)
* *Adj. (diet)	−0.42 (0.002)	−0.40 (0.002)	0.05 (0.167)	−0.45 (<0.001)
* *Adj. (full model)	−0.52 (<0.001)	−0.39 (0.001)	0.23 (0.100)	−0.45 (<0.001)
* Odoribacter splanchnicus*	−0.48 (<0.001)	−0.30 (0.016)	0.12 (0.596)	−0.21 (0.072)
* *Adj. (age and body fat)	−0.53 (<0.001)	−0.26 (0.019)	0.17 (0.346)	−0.17 (0.058)
* Roseburia inulinivorans*	−0.24 (0.044)	−0.43 (<0.001)	−0.14 (0.801)	−0.35 (0.006)
* *Adj. (age and body fat)	−0.31 (0.040)	−0.41 (0.001)	−0.11 (0.977)	−0.33 (0.005)
				
*Associated with an unhealthy lipid metabolism*				
* Catenibacterium mitsuokai*	0.16 (0.746)	0.45 (0.004)	−0.17 (0.986)	0.43 (0.005)
* *Adj. (age and body fat)	0.04 (0.639)	0.39 (0.007)	−0.09 (0.805)	0.37 (0.008)
* *Adj. (diet)	0.17 (0.898)	0.43 (0.008)	−0.24 (0.817)	0.42 (0.010)
* *Adj. (full model)	0.06 (0.747)	0.36 (0.012)	−0.19 (0.706)	0.35 (0.012)
* Holdemanella biformis*	0.27 (0.030)	0.49 (<0.001)	0.14 (0.935)	0.44 (<0.001)
* *Adj. (age and body fat)	0.27 (0.082)	0.46 (<0.001)	0.12 (0.779)	0.41 (<0.001)
* *Adj. (diet)	0.28 (0.099)	0.49 (<0.001)	0.18 (0.960)	0.43 (0.003)
* *Adj. (full model)	0.27 (0.183)	0.46 (<0.001)	0.06 (0.762)	0.40 (0.003)

Abbreviations: HDL-C, high-density lipoprotein cholesterol; LDL-C, low-density lipoprotein cholesterol; TAG, triglycerides. Correlations are presented as Pearson's *r* with *P*-values in parentheses, with and without adjustment (adj.) for age, body fat, and when relevant; the dietary component with the strongest impact (Supplementary Table S4). Full models are adjusted for age, body fat and diet.

## Notes

LKB, AA and LHL have as employees at Department of Nutrition, Exercise and Sports, University of Copenhagen received funding for research from Arla Foods amba and the Danish Dairy Board. AA is a consultant or member of advisory boards for: BioCare Copenhagen, DK; Basic Research, USA; Global Dairy Platform, USA; S-Biotek, DK. The remaining authors declare no conflict of interest.
