# Supplementary material for: Specific gut microbiota features and metabolic markers in postmenopausal women with obesity
Source: Nutr Diabetes. 2015 Jun 15;5(6):e159–. doi: 10.1038/nutd.2015.9 (PMC4491860; doi:10.1038/nutd.2015.9)
Supplement: Supplementary Table 1 [file nutd20159x3.doc]

Supplementary Table 1 Gut bacteria associated with insulin resistance

| MGS | Glucose (F) | | | Glucose  (AUC) | | Insulin  (F) | Insulin  (AUC) | | | | C-peptide  (F) | | | C-peptide  (AUC) | | HOMA-IR | | Matsudas index | | |
| --- | --- | --- | --- | --- | --- | --- | --- | --- | --- | --- | --- | --- | --- | --- | --- | --- | --- | --- | --- | --- |
| Gut bacteria negatively correlated with insulin resistance | | | | | | | | | | | | | | | | | | | | |
| Species | | | | | | | | | | | | | | | | | | | | |
| *Bacteroides faecis* | | -0.40 (0.003) | | -0.39 (0.004) | | -0.53 (<0.001) | -0.47 (<0.001) | | | | -0.57 (<0.001) | | | -0.48 (<0.001) | -0.55 (<0.001) | | | 0.52 (<0.001) | | |
| *Bifidobacterium longum* | | -0.54 (<0.001) | | -0.45 (<0.001) | | -0.30 (0.028) | -0.30  (0.027) | | | | -0.27 (0.022) | | | -0.28 (0.040) | -0.40 (0.003) | | | 0.40 (0.003) | | |
| *Dorea longicatena* | | -0.49 (<0.001) | | -0.42 (0.002) | | -0.19 (0.170) | -0.12  (0.402) | | | | -0.16 (0.258) | | | -0.12 (0.397) | -0.27 (0.055) | | | 0.25 (0.070) | | |
| *Faecalibacterium prausnitzii* *A2-165* | | -0.37 (0.007) | | -0.41 (0.003) | | -0.34 (0.012) | -0.30  (0.031) | | | | -0.31 (0.023) | | | -0.28 (0.042) | -0.39 (0.004) | | | 0.37 (0.007) | | |
| *Intestinibacter bartlettii* | | -0.41 (0.002) | | -0.48 (<0.001) | | -0.39 (0.004) | -0.38  (0.005) | | | | -0.38 (0.007) | | | -0.35 (0.010) | -0.43 (0.002) | | | 0.46 (<0.001) | | |
| Genus | | | | | | | | | | | | | | | | | | | | |
| *Acholeplasma sp. CAG:878* | -0.67 (<0.001) | | | -0.51 (<0.001) | | -0.50 (<0.001) | -0.38 (0.005) | | | | -0.44 (0.001) | | | -0.33 (0.018) | -0.58  (<0.001) | | | 0.54  (<0.001) | | |
| *Clostridium sp. CAG:492* | -0.47 (<0.001) | | | -0.50 (<0.001) | | -0.52 (<0.001) | -0.47 (<0.001) | | | | -0.47 (<0.001) | | | -0.43 (0.001) | -0.56 (<0.001) | | | 0.55 (<0.001) | | |
| *Eubacterium sp. CAG:252* | -0.47  (<0.001) | | | -0.58  (<0.001) | | -0.48  (<0.001) | -0.47 (<0.001) | | | | -0.60  (<0.001) | | | -0.60  (<0.001) | -0.51 (<0.001) | | | 0.51  (<0.001) | | |
| *Faecalibacterium sp.* | -0.31 (0.022) | | | -0.27 (0.050) | | -0.44 (0.001) | -0.34  (0.013) | | | | -0.40 (0.003) | | | -0.28 (0.046) | -0.46 (<0.001) | | | 0.41 (0.002) | | |
| Family | | | | | | | | | | | | | | | | | | | | |
| Lachnospiraceae | | -0.30 (0.029) | | -0.33 (0.015) | -0.48 (<0.001) | | -0.51 (<0.001) | | | | -0.48 (<0.001) | | | -0.46 (<0.001) | | -0.48 (<0.001) | | 0.51 (<0.001) | | |
| Lachnospiraceae | | -0.20 (0.153) | | -0.35 (0.009) | | -0.29 (0.033) | -0.35 (0.011) | | | | -0.24 (0.083) | | | -0.31 (0.027) | | -0.29 (0.035) | | 0.30 (0.027) | | |
| Porphyromonadaceae | | -0.43 (0.001) | | -0.44 (<0.001) | | -0.34 (0.013) | -0.31 (0.026) | | | | -0.27 (0.047) | | | -0.28 (0.044) | | -0.40 (0.003) | | 0.39 (0.004) | | |
| Order | | | | | | | | | | | | | | | | | | | | |
| Clostridiales | -0.25 (0.069) | | | -0.30 (0.029) | | -0.34 (0.012) | -0.46 (<0.001) | | | | -0.35 (0.010) | | | -0.41 (0.003) | | -0.35 (0.010) | | 0.41 (0.002) | | |
| Clostridiales | -0.18 (0.196) | | | -0.27 (0.047) | | -0.32 (0.020) | -0.38 (0.005) | | | | -0.32 (0.021) | | | -0.44 (0.001) | | -0.31 (0.022) | | 0.36 (0.008) | | |
| Phylum | | | | | | | | | | | | | | | | | | | | |
| Firmicutes | -0.48 (<0.001) | | | -0.60 (<0.001) | | -0.37 (0.006) | -0.40 (0.003) | | | | -0.36 (0.007) | | | -0.43 (0.001) | | -0.44  (0.001) | | 0.45 (<0.001) | | |
| Firmicutes | -0.36 (0.009) | | | -0.53 (<0.001) | | -0.26 (0.062) | -0.29 (0.035) | | | | -0.20 (0.155) | | | -0.27 (0.056) | | -0.30  (0.030) | | 0.34 (0.013) | | |
| Gut bacteria positively correlated with insulin resistance | | | | | | | | | | | | | | | | | | | | |
| Species | | | | | | | | | | | | | | | | | | | |  |
| *Bilophila wadsworthia* | 0.12 (0.393) | | 0.30 (0.028) | | 0.39 (0.004) | | | 0.29 (0.035) | | 0.39 (0.004) | | | 0.28 (0.043) | | | | 0.36 (0.009) | | -0.31 (0.023) | |
| *Clostridium*  *bolteae* | 0.43  (0.001) | | 0.54 (<0.001) | | 0.50 (<0.001) | | | 0.42 (0.002) | | 0.37 (0.006) | | | 0.38 (0.006) | | | | 0.54 (<0.001) | | -0.52 (<0.001) | |
| *Eubacterium*  *ramulus* | 0.44 (0.001) | | 0.45 (<0.001) | | 0.42 (0.002) | | | 0.35 (0.010) | | 0.35 (0.011) | | | 0.27 (0.049) | | | | 0.47 (<0.001) | | -0.46 (<0.001) | |
| *Ruminococcus torques* | 0.24 (0.078) | | 0.34 (0.013) | | 0.47 (<0.001) | | | 0.44 (0.001) | | 0.50 (<0.001) | | | 0.46 (<0.001) | | | | 0.47 (<0.001) | | -0.48 (<0.001) | |
| Genus | | | | | | | | | | | | | | | | | | | | |
| *Blautia* | 0.53 (<0.001) | | 0.38 (0.005) | | 0.50 (<0.001) | | 0.44 (0.001) | | | 0.43 (0.001) | | | 0.36 (0.010) | | | | 0.55 (<0.001) | | -0.54 (<0.001) | |
| *Clostridium* | 0.40 (0.003) | | 0.43 (0.002) | | 0.28 (0.046) | | 0.12 (0.392) | | | 0.21 (0.137) | | | 0.09 (0.530) | | | | 0.32 (0.021) | | -0.26 (0.063) | |
| *Clostridium* | 0.39 (0.004) | | 0.35 (0.011) | | 0.17 (0.222) | | 0.16 (0.243) | | | 0.15 (0.283) | | | 0.25 (0.074) | | | | 0.20 (0.145) | | -0.23 (0.091) | |
| *Clostridium sp. CAG:58* | 0.36 (0.008) | | 0.49 (<0.001) | | 0.58 (<0.001) | | | 0.51 (<0.001) | | 0.54 (<0.001) | | | 0.55 (<0.001) | | | | 0.55 (<0.001) | | -0.55 (<0.001) | |
| *Clostridium sp.CAG:91* | 0.38 (0.006) | | 0.23 (0.102) | | 0.47 (<0.001) | | | 0.33 (0.017) | | 0.37 (0.006) | | | 0.22 (0.123) | | | | 0.48 (<0.001) | | -0.44 (0.001) | |
| *Dorea sp. CAG:105* | 0.29 (0.037) | | 0.28 (0.040) | | 0.63  (<0.001) | | | 0.50 (<0.001) | | 0.56 (<0.001) | | | 0.39 (0.004) | | | | 0.60 (<0.001) | | -0.57 (<0.001) | |
| Family | | | | | | | | | | | | | | | | | | | | |
| Order | | | | | | | | | | | | | | | | | | | | |
| Clostridiales | 0.36 (0.009) | | 0.54 (<0.001) | | 0.39 (0.004) | | 0.46 (<0.001) | | 0.43 (0.001) | | | 0.55 (<0.001) | | | 0.42 (0.002) | | | -0.45 (<0.001) | | |
| Phylum | | | | | | | | | | | | | | | | | | | | |
| Firmicutes | 0.38 (0.005) | | 0.28 (0.039) | | 0.53 (<0.001) | | 0.53 (<0.001) | | 0.47 (<0.001) | | | 0.42 (0.002) | | | 0.53 (<0.001) | | | -0.55 (<0.001) | | |

Correlations are reported by Spearman's Rho (r) and P-values are given in parentheses. AUC, area-under-the-curve; F, fasting; HOMA-IR, homeostatic model assessment of insulin resistance; MGS, metagenomic species.
