# Supplementary material for: Specific gut microbiota features and metabolic markers in postmenopausal women with obesity
Source: Nutr Diabetes. 2015 Jun 15;5(6):e159–. doi: 10.1038/nutd.2015.9 (PMC4491860; doi:10.1038/nutd.2015.9)
Supplement: Supplementary Table 2 [file nutd20159x4.doc]

Supplementary Table 2 Gut bacteria associated with inflammatory markers and liver enzymes

| MGS | | LBP | | HsCRP | | WBC | | CD14 | | AST | | ALT |
| --- | --- | --- | --- | --- | --- | --- | --- | --- | --- | --- | --- | --- |
| Gut bacteria negatively correlated with inflammatory markers and liver enzymes | | | | | | | | | | | | |
| Species | | | | | | | | | | | | |
| *Bacteroides pectinophilus* | | 0.00  (0.999) | | 0.12  (0.399) | | -0.41  (0.003) | | 0.11  (0.446) | | -0.42  (0.002) | | -0.24  (0.080) |
| Genus | | | | | | | | | | | | |
| *Clostridium* | | -0.17  (0.234) | | 0.06  (0.691) | | -0.28  (0.043) | | -0.15  (0.269) | | -0.45  (<0.001) | | -0.46  (<0.001) |
| *Eggerthella sp. CAG:209* | | -0.08  (0.586) | | -0.14  (0.324) | | -0.35  (0.010) | | 0.11  (0.439) | | -0.32  (0.019) | | -0.52  (<0.001) |
| *Eubacterium sp. CAG:252* | | -0.09  (0.541) | | 0.02  (0.915) | | -0.25  (0.073) | | 0.06  (0.684) | | -0.46  (<0.001) | | -0.39  (0.004) |
| *Firmicutes bacterium sp.*  *CAG:95* | | -0.05  (0.723) | | -0.08  (0.592) | | -0.33  (0.016) | | -0.05  (0.714) | | -0.53  (<0.001) | | -0.40  (0.003) |
| *Roseburia sp. CAG:100* | | -0.09  (0.540) | | 0.06  (0.656) | | -0.15  (0.274) | | 0.03  (0.809) | | -0.48  (<0.001) | | -0.42  (0.002) |
| *Ruminococcus* | | -0.19  (0.166) | | -0.49  (<0.001) | | -0.16  (0.245) | | 0.05  (0.703) | | 0.03  (0.821) | | 0.00  (0.979) |
| Family | | | | | | | | | | | | |
| Lachnospiraceae | | -0.27  (0.047) | | -0.48  (<0.001) | | -0.36  (0.009) | | -0.03  (0.840) | | -0.06  (0.691) | | -0.34  (0.012) |
| Lachnospiraceae | | -0.19  (0.169) | | 0.00  (0.999) | | -0.19  (0.168) | | 0.06  (0.645) | | -0.25  (0.073) | | -0.42  (0.002) |
| Order | | | | | | | | | | | | |
| Clostridiales | | -0.05  (0.712) | | -0.25  (0.075) | | -0.29  (0.036) | | -0.05  (0.713) | | -0.34  (0.013) | | -0.45  (<0.001) |
| Clostridiales | | -0.02  (0.896) | | -0.17  (0.216) | | -0.25  (0.074) | | -0.09  (0.528) | | -0.36  (0.008) | | -0.49  (<0.001) |
| Clostridiales | | -0.06  (0.679) | | -0.22  (0.120) | | -0.36  (0.008) | | -0.09  (0.541) | | -0.30  (0.029) | | -0.41  (0.002) |
| Phylum | | | | | | | | | | | | |
| Firmicutes | | -0.07  (0.599) | | -0.28  (0.046) | | -0.32  (0.021) | | -0.11  (0.413) | | -0.42  (0.002) | | -0.26  (0.058) |
| Gut bacteria positively correlated with inflammatory markers and liver enzymes | | | | | | | | | | | | |
| Species | | | | | | | | | | | | |
| *Bilophila wadsworthia* | 0.12  (0.404) | | 0.03  (0.810) | | 0.24  (0.080) | | 0.10  (0.494) | | 0.39  (0.004) | | 0.46 (<0.001) | |
| *Clostridium bolteae* | 0.16  (0.245) | | 0.03  (0.808) | | 0.33  (0.016) | | -0.14 (0.321) | | 0.45 (<0.001) | | 0.70 (<0.001) | |
| *Dorea formicigenerans* | 0.27  (0.055) | | 0.41  (0.002) | | -0.05 (0.726) | | 0.44  (<0.001) | | -0.11  (0.448) | | 0.04  (0.760) | |
| *Faecalibacterium*  *prausnitzii* *SL3/3* | 0.16  (0.267) | | 0.50  (<0.001) | | 0.22  (0.108) | | 0.14  (0.320) | | -0.12  (0.396) | | 0.00  (0.998) | |
| *Roseburia hominis* | 0.32  (0.019) | | 0.47  (<0.001) | | -0.09 (0.538) | | 0.16  (0.265) | | -0.15  (0.295) | | -0.12  (0.412) | |
| Genus | | | | | | | | | | | | |
| *Alistipes* *sp. HGB5* | 0.48 (<0.001) | | 0.16  (0.248) | | 0.07  (0.624) | | 0.16  (0.239) | | 0.11  (0.429) | | 0.14  (0.325) | |
| *Anaerotruncus sp.*  *CAG:528* | -0.19  (0.159) | | -0.29 (0.035) | | -0.18 (0.205) | | -0.20 (0.154) | | -0.24  (0.083) | | -0.31  (0.023) | |
| *Clostridium* | 0.04  (0.761) | | 0.00  (0.983) | | 0.09  (0.517) | | 0.31  (0.026) | | 0.67 (<0.001) | | 0.44 (<0.001) | |
| *Clostridium sp. CAG:43* | 0.34  (0.012) | | 0.48  (<0.001) | | 0.14  (0.309) | | 0.21  (0.127) | | 0.09  (0.518) | | 0.17  (0.231) | |
| Order | | | | | | | | | | | | |
| Clostridiales | 0.48 (<0.001) | | 0.10  (0.492) | | 0.12  (0.404) | | 0.22  (0.119) | | 0.09  (0.530) | | 0.10  (0.472) | |

Correlations are reported by Spearman's Rho (r) and P-values are given in parentheses. ALT, alanine aminotransferase; AST, aspartate aminotransferase; CD14, cluster of differentiation 14; hsCRP, high sensitive C-reactive protein; LBP, lipopolysaccharide-binding protein; MGS, metagenomic species; WBC, white blood cell count.
