# Supplementary material for: Specific gut microbiota features and metabolic markers in postmenopausal women with obesity
Source: Nutr Diabetes. 2015 Jun 15;5(6):e159–. doi: 10.1038/nutd.2015.9 (PMC4491860; doi:10.1038/nutd.2015.9)
Supplement: Supplementary Table 3 [file nutd20159x5.doc]

Supplementary Table 3 Gut bacteria associated with lipid metabolism

| MGS | TAG | Cholesterol | HDL-C | LDL-C | FFA |
| --- | --- | --- | --- | --- | --- |
| Species | | | | | |
| *Akkermansia muciniphila* | -0.14  (0.302) | -0.57  (<0.001) | 0.01  (0.952) | -0.56  (<0.001) | 0.06  (0.695) |
| *Bacteroides cellulosilyticus* | -0.34  (0.012) | -0.50  (<0.001) | -0.08  (0.559) | -0.46  (<0.001) | -0.08  (0.569) |
| *Bacteroides nordii* | -0.21  (0.124) | -0.59  (<0.001) | 0.06  (0.653) | -0.59  (<0.001) | -0.10  (0.456) |
| *Bacteroides pectinophilus* | -0.50  (<0.001) | -0.53  (<0.001) | 0.18  (0.185) | -0.55  (<0.001) | 0.06  (0.671) |
| *Bifidobacterium* *longum* | -0.21  (0.136) | -0.01  (0.919) | -0.06  (0.683) | 0.02  (0.864) | -0.42  (0.002) |
| *Catenibacterium mitsuokai* | 0.31  (0.026) | 0.77  (<0.001) | 0.06  (0.657) | 0.76  (<0.001) | 0.00  (0.974) |
| *Faecalibacterium prausnitzii*  A2-165 | -0.02  (0.878) | -0.24  (0.089) | 0.05  (0.701) | -0.28  (0.044) | 0.07  (0.627) |
| *Holdemanella biformis* | 0.30  (0.032) | 0.53  (<0.001) | 0.01  (0.921) | 0.52  (<0.001) | -0.08  (0.580) |
| *Odoribacter splanchnicus* | -0.47  (<0.001) | -0.27  (0.048) | 0.24  (0.079) | -0.27  (0.052) | -0.01  (0.916) |
| *Roseburia inulinivorans* | -0.18  (0.201) | -0.56  (<0.001) | 0.03  (0.857) | -0.50  (<0.001) | 0.06  (0.692) |
| Genus |  |  |  |  |  |
| *Anaerotruncus sp. CAG:528* | -0.45  (0.001) | -0.12  (0.385) | 0.57  (<0.001) | -0.25  (0.067) | -0.08  (0.581) |
| *Bacteroidales bacterium ph8* | -0.19  (0.175) | -0.49  (<0.001) | -0.02  (0.892) | -0.43  (0.001) | 0.16  (0.264) |
| *Collinsella sp.* | -0.32  (0.019) | -0.48  (<0.001) | 0.12  (0.409) | -0.48  (<0.001) | -0.07  (0.616) |
| *Eubacterium* | -0.31  (0.026) | 0.05  (0.696) | 0.46  (<0.001) | -0.05  (0.699) | -0.15  (0.288) |
| *Prevotella sp. CAG:386* | 0.14  (0.304) | 0.48  (<0.001) | 0.13  (0.354) | 0.42  (0.002) | 0.11  (0.449) |
| *Ruminococcus sp. CAG:60* | -0.37  (0.006) | -0.35  (0.010) | 0.47  (<0.001) | -0.44  (<0.001) | 0.02  (0.900) |
| *Tannerella* sp. *6 1 58FAA CT1* | -0.25  (0.066) | -0.54  (<0.001) | 0.15  (0.290) | -0.50  (<0.001) | -0.07  (0.637) |
| *Veillonella*  *sp. 6 1 27* | 0.06  (0.665) | 0.04  (0.763) | -0.53  (<0.001) | 0.18  (0.201) | 0.11  (0.441) |
| Order | | | | | |
| Clostridiales | -0.23  (0.094) | -0.53  (<0.001) | 0.09  (0.543) | -0.54  (<0.001) | 0.14  (0.328) |
| Clostridiales | -0.51 (<0.001) | -0.20  (0.149) | 0.22  (0.115) | -0.15  (0.288) | -0.49  (<0.001) |
| Clostridiales | -0.18  (0.192) | -0.12  (0.404) | 0.09  (0.545) | -0.10  (0.471) | -0.54  (<0.001) |
| Clostridiales | -0.35  (0.011) | -0.21  (0.123) | 0.16  (0.241) | -0.21  (0.124) | 0.01  (0.940) |
| Clostridiales | 0.42  (0.002) | 0.02  (0.876) | -0.15  (0.289) | 0.00  (0.989) | 0.27  (0.052) |
| Phylum | | | | | |
| Firmicutes | -0.28  (0.043) | -0.47  (<0.001) | 0.00  (0.980) | -0.41  (0.002) | 0.19  (0.183) |

Correlations are reported by Spearman's Rho (r) and P-values are given in parentheses. FFAs, free fatty acids; HDL-C, high-density lipoprotein cholesterol; hsCRP, high sensitive C-reactive protein; LDL-C, low-density lipoprotein cholesterol; MGS, metagenomic species; TAG, triglycerides.
