# Supplementary material for: Specific gut microbiota features and metabolic markers in postmenopausal women with obesity
Source: Nutr Diabetes. 2015 Jun 15;5(6):e159–. doi: 10.1038/nutd.2015.9 (PMC4491860; doi:10.1038/nutd.2015.9)
Supplement: Supplementary Table 4 [file nutd20159x6.doc]

Supplementary Table 4 Correlations between metabolic-associated bacterial species, bacterial diversity and dietary intake

| Species | Energy (kJ/day) | Protein (E%) | CHO (E%) | DF (g/day) | Fat (E%) |
| --- | --- | --- | --- | --- | --- |
| Associated with several beneficial metabolic traits | | | | | |
| Bacterial gene count | -0.25 (0.078) | 0.29 (0.037) | -0.02 (0.885) | 0.02 (0.918) | -0.18 (0.20) |
| Exponential Shannon index | -0.12 (0.401) | 0.24 (0.085) | -0.03 (0.810) | 0.06 (0.698) | -0.09 (0.523) |
| *Bacteroides faecis* | 0.10 (0.466) | -0.12 (0.408) | 0.28 (0.043) | 0.06 (0.696) | -0.21 (0.134) |
| *Bacteroides pectinophilus* | -0.13 (0.367) | 0.29 (0.035) | 0.17 (0.225) | 0.12 (0.385) | -0.32 (0.019) |
| *Bifidobacterium longum* | 0.22 (0.125) | -0.09 (0.513) | 0.42 (0.002) | 0.39 (0.004) | -0.24 (0.093) |
| *Faecalibacterium prausnitzii A2-165* | -0.33 (0.017) | 0.24 (0.090) | 0.20 (0.155) | 0.10 (0.505) | -0.47 (<0.001) |
| Associated with several non-beneficial metabolic traits | | | | | |
| *Clostridium bolteae* | 0.16 (0.248) | 0.01 (0.971) | -0.31 (0.025) | -0.32 (0.022) | 0.35 (0.011) |
| *Bilophila wadsworthia* | 0.23 (0.095) | -0.15 (0.290) | 0.03 (0.831) | 0.10 (0.465) | 0.069 (0.629) |
| Negatively associated with insulin resistance | | | | | |
| *Dorea longicatena* | -0.28 (0.046) | 0.23 (0.101) | 0.19 (0.17) | -0.15 (0.301) | -0.15 (0.277) |
| *Intestinibacter bartlettii* | 0.03 (0.813) | 0.04 (0.774) | 0.34 (0.014) | 0.24 (0.089) | -0.20 (0.151) |
| Positively associated with insulin resistance | | | | | |
| *Ruminococcus torques* | -0.05 (0.717) | 0.17 (0.237) | -0.16 (0.270) | -0.23 (0.098) | 0.26 (0.066) |
| Positively associated with inflammation | | | | | |
| *Dorea formicigenerans* | -0.14 (0.308) | 0.27 (0.057) | -0.07 (0.640) | -0.04 (0.758) | 0.02 (0.918) |
| *Faecalibacterium prausnitzii SL3/3* | -0.09 (0.544) | 0.29 (0.039) | -0.03 (0.849) | 0.05 (0.711) | -0.09 (0.535) |
| Associated with a healthy lipid profile | | | | | |
| *Akkermansia muciniphila* | -0.06 (0.667) | -0.08 (0.572) | 0.23 (0.104) | 0.13 (0.372) | -0.28 (0.044) |
| *Bacteroides cellulosilyticus* | 0.09 (0.511) | -0.05 (0.718) | 0.21 (0.139) | 0.30 (0.034) | -0.13 (0.346) |
| Associated with an unhealthy lipid profile | | | | | |
| *Catenibacterium mitsuokai* | -0.19 (0.168) | 0.12 (0.406) | -0.25 (0.08) | -0.16 (0.256) | 0.17 (0.226) |
| *Holdemanella biformis* | -0.19 (0.185) | 0.23 (0.100) | -0.34 (0.014) | -0.18 (0.201) | 0.09 (0.521) |

Correlations are reported by Spearman's Rho (r) and P-values are given in parentheses. CHO, carbohydrates; DF, dietary fibres; E%, energy percentage.
